# Sex-Related Effect of Chronic Doses of Warfarin and Menadione on *Drosophila melanogaster*

**DOI:** 10.3390/ijms27136026

**Published:** 2026-07-04

**Authors:** Anna Lavrenova, Maria Kozlova, Oleg Klychnikov, Lidia Nefedova

**Affiliations:** Faculty of Biology, M.V. Lomonosov Moscow State University, 119991 Moscow, Russia; anigd_1999@mail.ru (A.L.); kozlovamary2002@gmail.com (M.K.); oklych@yahoo.co.uk (O.K.)

**Keywords:** *Drosophila melanogaster*, warfarin, menadione, vitamin K, lifespan, ATP hydrolysis, mitochondrial stress

## Abstract

Vitamin K is a well-established cofactor for γ-glutamyl carboxylase in vertebrates, yet its physiological functions in insects remain poorly understood. Warfarin, a vitamin K antagonist, inhibits vitamin K epoxide reductase, thereby blocking the vitamin K cycle. We have previously demonstrated that, in *Drosophila melanogaster*, vitamin K3 (menadione sodium bisulfite, MSB) is converted into endogenous vitamin K2, and that warfarin interferes with this cycle, leading to reduced oxidative stress. In the present study, we investigated the effects of chronic exposure to MSB (3.5 mM) and/or warfarin (1 mM and 10 mM) during larval development on adult survival, lifespan, mitochondrial ATP hydrolysis rate, and the transcriptional expression of target genes. Warfarin at 1 mM did not affect development or egg laying, whereas 10 mM caused significant postembryonic lethality. Transcriptional analysis revealed marked sex-specific effects. In females, warfarin downregulated the *levy* gene (encoding a complex IV subunit) and the *ATPsynB* gene (encoding an ATP synthase subunit B), while MSB suppressed cell cycle genes (*cmet*, *sti*, *mcm2*). In contrast, males exhibited upregulation of *COQ7* (encoding a key enzyme in ubiquinone biosynthesis) in response to warfarin and increased *sti* expression in response to MSB, suggesting a more robust compensatory response. Notably, the combined application of MSB and warfarin increased mitochondrial ATP hydrolysis, thereby offsetting the negative physiological effects despite persistent transcriptional suppression. In females, either MSB or warfarin alone significantly reduced median lifespan; however, when applied together, female lifespan was restored to control levels. In males, the effects were considerably weaker. Thus, this study reveals a novel, sex-specific interaction between vitamin K and its antagonist in *D. melanogaster*, providing new insights into mitochondrial adaptation and sexual dimorphism in metabolic stress responses.

## 1. Introduction

Vitamin K is one of the important compounds for most living organisms [1]. There are three forms of vitamin K: K1 (phylloquinone), K2 (menaquinone), and K3 (menadione). Phylloquinone is the native form of vitamin K in plants, menaquinone in animals and bacteria, and menadione is a synthetic analog of vitamin K. In medical practice, menadione sodium bisulfite (MSB) is used, which, unlike menadione, is soluble in water.

In plants and bacteria, vitamin K acts as an electron carrier in the electron transport chain, while, in humans and other vertebrates, vitamin K is a cofactor of the enzyme gamma-glutamyl carboxylase (GGC) [2]. During the gamma-carboxylation reaction, vitamin K is oxidized to an epoxide and then reduced in two steps by the enzyme vitamin K oxidoreductase (VKOR), first to quinone and then to hydroquinone, which is the cofactor of the gamma-carboxylation reaction. The sequential processes of vitamin K oxidation and reduction are referred to as the vitamin K cycle [3].

It has been shown that a water-soluble salt of vitamin K3, menadione sodium bisulfite (MSB), in high concentrations can act as an inducer of oxidative stress, as it can stimulate the production of reactive oxygen species and leads to apoptosis [4]. MSB was shown to selectively accumulate superoxide anions directly within the mitochondria, leading to mitochondrial dysfunction and critical depletion of cellular ATP stores [5].

Warfarin, a coumarin derivative, is a vitamin K antagonist in vertebrates [6]. Despite the availability of numerous anticoagulants, warfarin is widely used due to its efficacy in the treatment of thromboembolic diseases. Like all coumarins, warfarin is an indirect anticoagulant and is therefore toxic to vertebrates at high doses. The toxic effect of warfarin is due to its binding to the VKOR enzyme and inhibiting the reduction of vitamin K epoxide; the reduced form is active and can be used by GGC. Various hypotheses have been proposed regarding the inhibition mechanism, but the process is still not fully understood. Some studies [7] have shown that warfarin acts on VKOR via competitive inhibition, while other data [8] suggest that warfarin inhibits VKOR via non-competitive inhibition. One of the more recently published studies [9] showed that the warfarin binding site may be located both inside and outside the vitamin K binding site, which explains the different data on the inhibition mechanism. As an anticoagulant, warfarin is often used in the therapy of venous thromboembolism caused by cancer [10]. However, in recent years, it has been largely replaced by low-molecular-weight heparins and direct oral anticoagulants due to the need for individualized dosing and consideration of drug interactions when using warfarin [11]. A recent study has shown that warfarin directly binds to the MAPK8 protein and affects its activity [12]. MAPK8 is a key stress kinase that is activated by oxidative stress, cytokines, and mitochondrial dysfunction.

*Drosophila melanogaster* is widely used as a model for studying metabolic processes and their disruption in humans. However, menadione is used in *D. melanogaster* experiments exclusively as an oxidizing agent that induces oxidative stress, because the role of vitamin K in its organism has not been studied, and warfarin has not been used previously. In our previous study, we demonstrated that MSB can be converted into vitamin K2 in *D. melanogaster*. Warfarin was found to block vitamin K reduction, thereby reducing the level of oxidative stress caused by high concentrations of vitamin K [13]. Furthermore, warfarin at the tested concentrations did not have the same toxic effect on flies as on vertebrates, as it does not exert an anticoagulant effect, but rather rescued flies from the toxic effects of vitamin K. Thus, *D. melanogaster* is the first invertebrate model system for studying the vitamin K cycle. A more in-depth investigation into the molecular mechanisms of vitamin K metabolism in Drosophila may provide a more profound understanding of its significance and functions in animals, including mammals. The aim of this work is to study the physiological, biochemical, and genetic aspects of the combined effects of MSB and warfarin on *D. melanogaster*.

## 2. Results

### 2.1. Study of the Effect of Warfarin on the Survival and Egg Laying of Drosophila melanogaster

**Figure 1:**
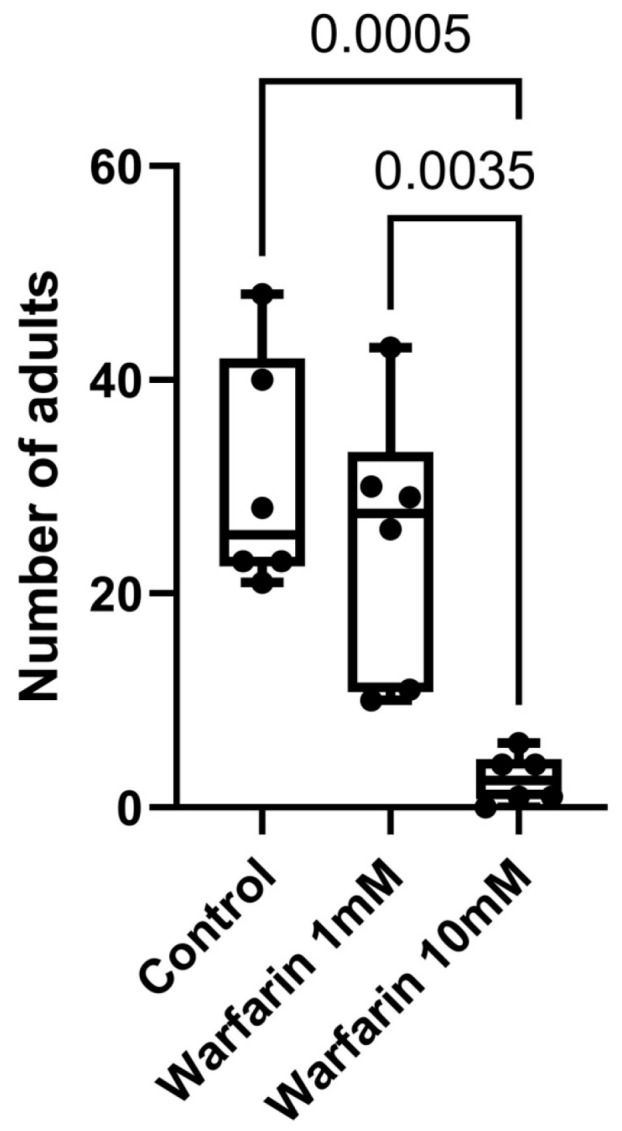
Number of adult flies after development on media with different concentrations of warfarin. Statistical analysis of data using one-way ANOVA test with Dunn’s post hoc test. Each dot indicates a sample.

Previously, we analyzed the effects of menadione sodium bisulfite (MSB) on adult survival and determined that MSB negatively affects survival of *D. melanogaster* from egg to adult [13]. To assess the effect of warfarin on *D. melanogaster* development, two-day-old males and females for laying eggs were placed on media containing warfarin at concentrations of 1 mM and 10 mM. We then counted the number of eclosed adults, as well as the number of non-eclosed larvae and pupae that died. On the medium with warfarin at a concentration of 1 mM, we found no statistically significant differences from the control group, whereas warfarin at a concentration of 10 mM significantly reduced survival and also caused mortality at the larval stage (Figure 1). Thus, we can conclude that, at a concentration of 10 mM, warfarin substantially increases embryonic and larval mortality, whereas 1 mM warfarin does not significantly affect fly development.

Next, it was necessary to verify that the low offspring count was not due to a reduced rate of egg laying on the warfarin-containing medium. It is known that, in some cases, *D. melanogaster* exhibits selectivity toward the media on which it lays eggs and may avoid laying eggs on a medium unsuitable for embryonic development [14]. We placed 10 females on different media and counted the number of eggs laid within 24 h. In a medium containing 10 mM warfarin, females laid 152 eggs. When exposed to 1 mM warfarin, they laid 113 eggs; meanwhile, in a control medium containing no warfarin, they laid 134 eggs. Based on these data, we can conclude that warfarin does not affect either the choice of laying medium or the egg laying itself. Warfarin at 10 mM does not reduce female fertility but rather reduces offspring survival (egg-to-imago). Thus, the toxic effect of warfarin is specifically postembryonic at high concentrations.

### 2.2. Analysis of the Effect of MSB and Warfarin on the ATP Hydrolysis Rate in D. melanogaster

**Figure 2:**
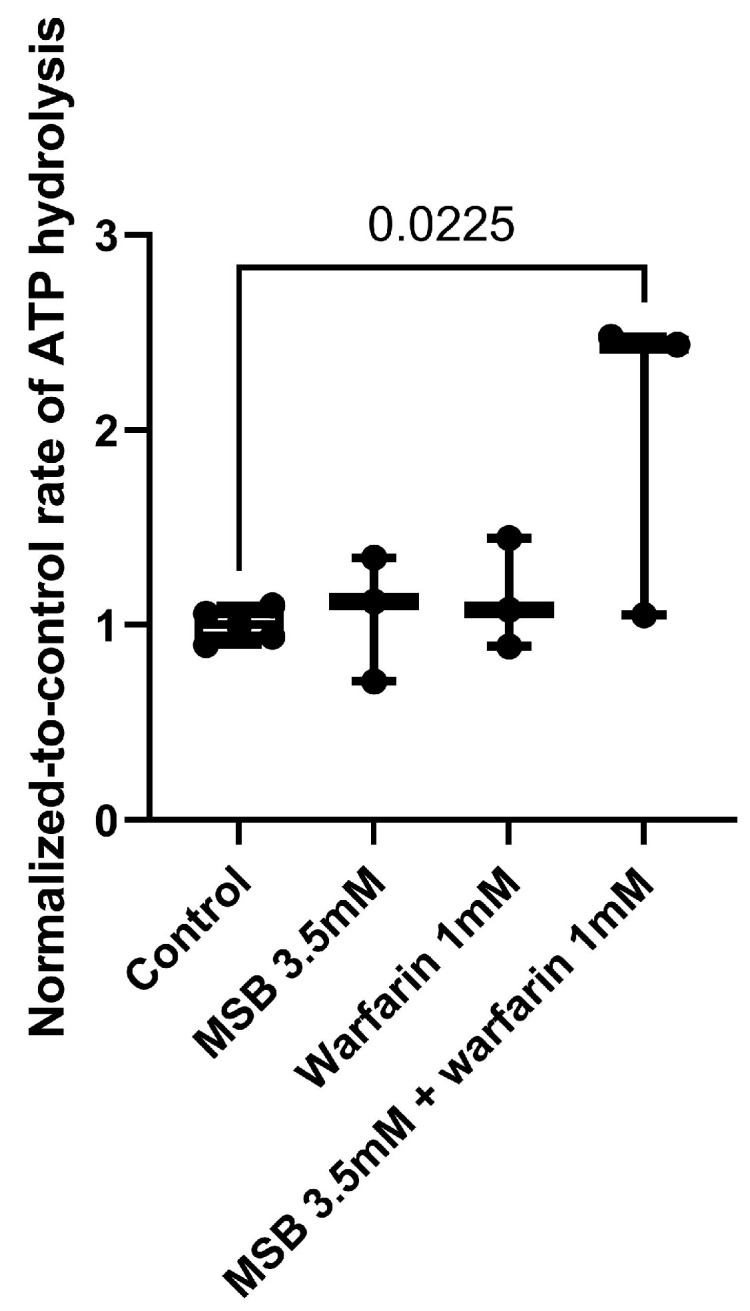
The relative rate of oligomycin-sensitive ATP hydrolysis by mitochondrial samples isolated from two-day-old *D. melanogaster*. Statistical analysis of data using one-way ANOVA test with Dunn’s post hoc test. Each dot indicates a sample.

It was found that not only MSB but also warfarin caused mitochondrial disfunction and reduced the cellular ATP levels leading to compromised viability [15]. We assessed the effect of exposure to MSB and warfarin during larval development on the ATP hydrolysis rate in mitochondria of two-day-old adults. Relative to control, the ATP hydrolysis rates are shown in Figure 2. The relative ATP hydrolysis rate in mitochondria of Drosophila exposed to the mixture of MSB and warfarin was higher than in the control and, unexpectedly, higher than in flies that developed on MSB or warfarin alone.

### 2.3. Analysis of the Expression Levels of Genes Involved in Key Cellular Processes That Are Targets of Menadione and Warfarin in D. melanogaster

**Figure 3:**
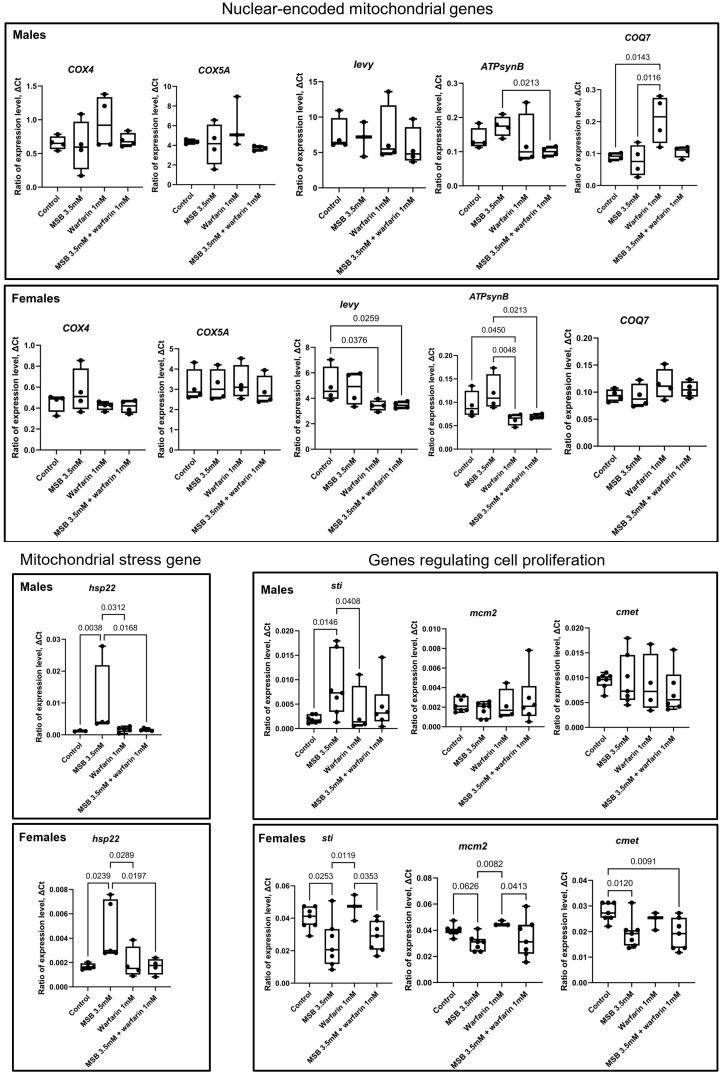
Relative expression levels of genes involved in key cellular processes in *D. melanogaster* under the action of MSB and warfarin. Statistical analysis of data using Kruskal–Wallis test with Dunn’s post hoc test. Each dot indicates a sample.

Since we found an effect of warfarin and MSB on ATP synthesis and content in *D. melanogaster*, we decided to examine the expression levels of genes that are potential targets of these compounds. To assess the direct effect on oxidative phosphorylation, considering the role of MSB as an electron acceptor and the involvement of warfarin in vitamin K metabolism, genes involved in the mitochondrial respiratory chain (*cox4*, *cox5a*, *levy* (*cox6a*)) and ubiquinone biogenesis (*COQ7*) were selected. The *ATPsynB* gene was included to analyze the efficiency of ATP synthesis. The chaperone *hsp22* gene transcription level was used as marker of mitochondrial stress. The genes *sti, mcm2* and *cmet* were used as genes regulating cell proliferation. Given that males and females respond differently to the delayed effects of MSB and warfarin, we performed transcriptional analysis separately for males and females (Figure 3).

When examining mitochondrial respiration and ATP synthase genes (*cox4*, *cox5a*, *levy*, *ATPsynB*), we found that genes encoding subunits of complex IV (cytochrome c oxidase)—*cox4* and *cox5a*—did not change their expression. The *levy* gene decreased in expression under the action of warfarin and a compound of MSB and warfarin, but only in females. The *ATPsynB* gene decreased in expression under the action of warfarin both in females and males under the action of warfarin and a compound of MSB and warfarin. We found that the expression level of the *COQ7* gene was statistically significantly increased following warfarin exposure in males but not in females. Thus, warfarin has a negative effect on certain mitochondrial genes; this effect depends on sex; and MSB cannot compensate for it.

Increased expression of *hsp22*, a marker gene of mitochondrial stress, was observed on the MSB-containing medium in both females and males. Warfarin mitigated the mitochondrial oxidative stress, which is consistent with results showing that it is not the action of MSB alone that causes the increase in ATP hydrolysis rate but rather its combination with warfarin.

Expression of the *sti, cmet* and *mcm2* genes, encoding proteins involved in DNA replication and the cell cycle, in females changed in a similar manner and was reduced under the action of MSB. MSB did not compensate in full measure for this effect. It is noteworthy that the reaction of the *sti* gene to MSB was opposite in males and females.

### 2.4. Assessment of the Effect of Warfarin and Menadione on the Lifespan of D. melanogaster

**Figure 4:**
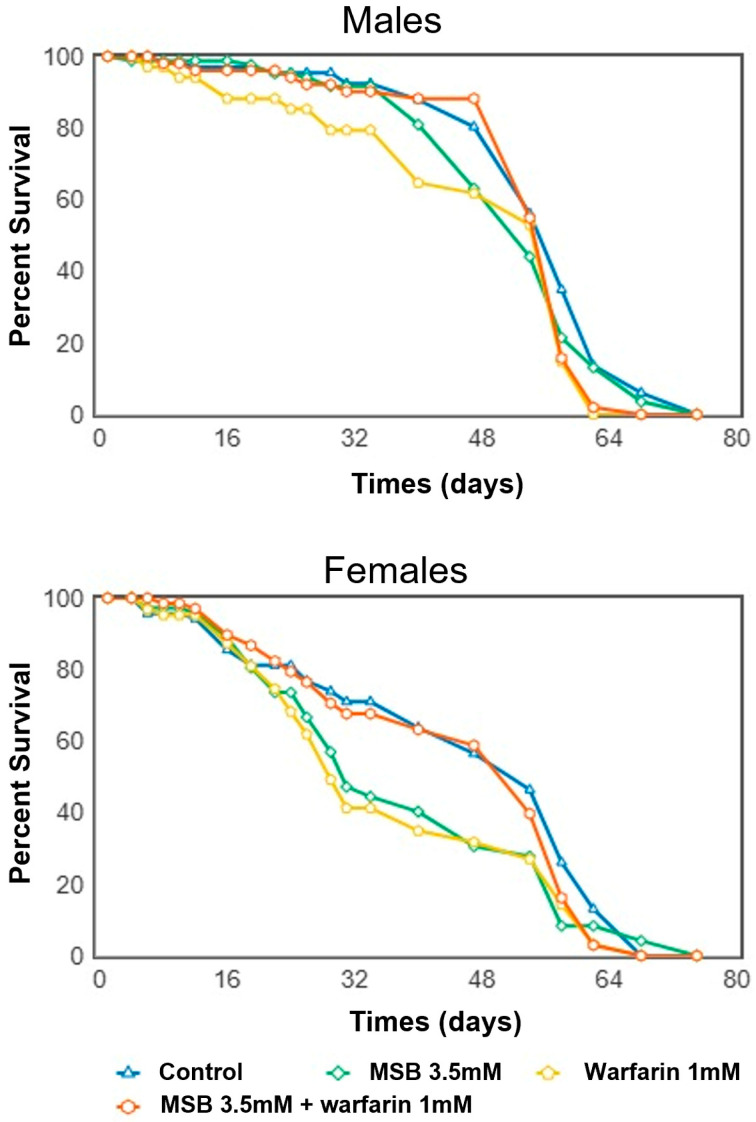
Delayed effects of warfarin and MSB on the lifespan of *D. melanogaster* males and females. Statistical analysis of data using log-rank test.

Previously, we analyzed the effects of MSB and warfarin on the survival of *D. melanogaster* larvae [13]. In the current study, we assessed the delayed effect of the previously determined doses of MSB (3.5 mM) and warfarin (1 mM), administered during larval development, on the lifespan of adults on the standard media (Figure 4; Table 1 and Table 2). As a result, we found that both MSB and warfarin acquired during development have a negative impact on the average lifespan of females, reducing q50 by approximately 40% (Table 3). In males, mean lifespan was slightly reduced after exposure to warfarin only (Table 4). It was found that, in females, the average lifespan decreased in response to exposure to either MSB or warfarin. Combined application of these substances during development leveled out the independent negative effect of each of them, but only in females.

## 3. Discussion

In our study, we found that 1 mM warfarin is not toxic to *D. melanogaster* females during egg laying and does not affect fly development from egg to adult; however, it has a delayed effect on the average lifespan of females, similar to MSB. The delayed effect of warfarin may be related to the inhibition of vitamin K reduction, which disrupts, possibly, the carboxylation of specific proteins.

The combination of MSB and warfarin leads to an increase in the ATP hydrolysis rate in mitochondria by F-ATPase and a paradoxical decrease in the expression of the *ATPsynB* and *levy* genes. Since the exposure was chronic, the increase in ATP hydrolysis rate in this case is not a stress response but rather an adaptation to the action of a ROS-generating agent together with blockade of the vitamin K reduction cycle. The fact that this is an adaptation is evidenced by the absence of a pronounced increase in ROS in the fly organism on the tested media, as we have previously shown [13]. Thus, flies during development adapted to MSB and successfully neutralized ROS. Under the combined action of MSB and warfarin, the latter apparently blocks the endogenous synthesis of ubiquinone-like vitamin K derivatives, while MSB can probably replace endogenous ubiquinone. In response, the cell activates protective F-ATPases in mitochondria that attempt to restore membrane potential and ionic homeostasis, which increases ATP hydrolysis. Oligomycin is a highly specific inhibitor of the mitochondrial F_0_F_1_-ATP synthase, acting by blocking the proton channel within the F_0_ domain. Its ability to inhibit ATP hydrolysis serves as a functional marker of an intact and coupled F_0_F_1_-ATPase in isolated mitochondrial preparations. We observed the effects of combining MSB and warfarin on F-ATPase activity in our samples, which led us to conclude that this ATP catabolism pathway is specifically affected in mitochondria.

At the same time, genes encoding subunits of complex IV (cytochrome c oxidase) did not change their expression upon exposure to MSB and warfarin, whereas the *levy* gene decreased in expression under the action of warfarin and MSB. The *levy* gene encodes a protein associated with complex IV and involved in the regulation of lifespan and electron transport [16].

Warfarin reduced *ATPsynB* gene transcription, indicating suppression of mitochondrial function. However, only under the combined action of warfarin and MSB, despite the persistent decrease in the expression of these genes (*levy* and *ATPsynB*), a high rate of ATP hydrolysis was observed. This can be explained by the fact that MSB compensates for the warfarin-induced deficiency of quinones in the electron transport chain. The decrease in *ATPsynB* expression upon warfarin exposure indicates a rearrangement of the energy apparatus. It has been described in the literature that reduced ATP synthase activity can increase lifespan in *D. melanogaster* [17].

We found that the expression level of the *COQ7* gene was statistically significantly increased following warfarin exposure in males. This may be explained by the fact that warfarin reduces the availability of reduced vitamin K. Therefore, the increase in *COQ7* gene transcription may occur as a compensatory cellular response to the presence of warfarin in the absence of exogenous MSB, and the cell enhances its own ubiquinone synthesis. It is noteworthy that an increase in *COQ7* expression levels was not observed in females under any of the treatments. This can be explained by the fact that basal levels of ubiquinone and respiratory chain activity may be lower in males than in females, which have a much higher metabolic demand due to egg production. When warfarin blocks the vitamin K-dependent pathway and creates a quinone deficiency, the ubiquinone pool in males becomes critically depleted. Therefore, males are compelled to upregulate *COQ7* transcription as a compensatory survival mechanism.

Increased expression of *hsp22* on the MSB-containing medium is a marker of mitochondrial oxidative stress in both females and males. It is localized in mitochondria, induced during aging, and is part of the unfolded protein response. The literature data indicate that its moderate overexpression extends *D. melanogaster* lifespan, whereas its absence or excess shortens it [18]. Warfarin mitigates mitochondrial oxidative stress, which is consistent with results showing that it is not the action of MSB alone that causes the increase in the ATP hydrolysis rate but rather its combination with warfarin.

The products of the *sti, cmet* and *mcm2* genes encode proteins involved in DNA replication and the cell cycle. As a ROS generator, MSB damages DNA, so the decreased expression of these genes is a classic sign of cell cycle arrest or suppressed proliferation. This explains why MSB is toxic to developing larvae. At the same time, it is known that increased ROS production, particularly due to reverse electron transport through respiratory complex I, extends the lifespan of adult *D. melanogaster* [19]. Reverse electron transport prevents pathogenesis caused by severe oxidative stress, underscoring the importance of the site of ROS production in signaling.

Taken together, these data indicate that females on the medium containing the mixture of MSB and warfarin live normally because the negative effects of MSB, manifested in the suppression of *cmet*, *mcm2*, and *sti* transcription, and of warfarin, manifested in the suppression of *levy* and *ATPsynB*, are mutually compensated at the physiological level. Furthermore, the reduced expression of *levy* and *ATPsynB* is compensated by increased ATPase activity. Notably, among all the genes studied, only *hsp22*, *ATPsynB*, *COQ7*, and *sti* demonstrated significant expression changes in males. This is likely due to sex-specific differences in energy and redox metabolism as well as in aging rates.

**Figure 5:**
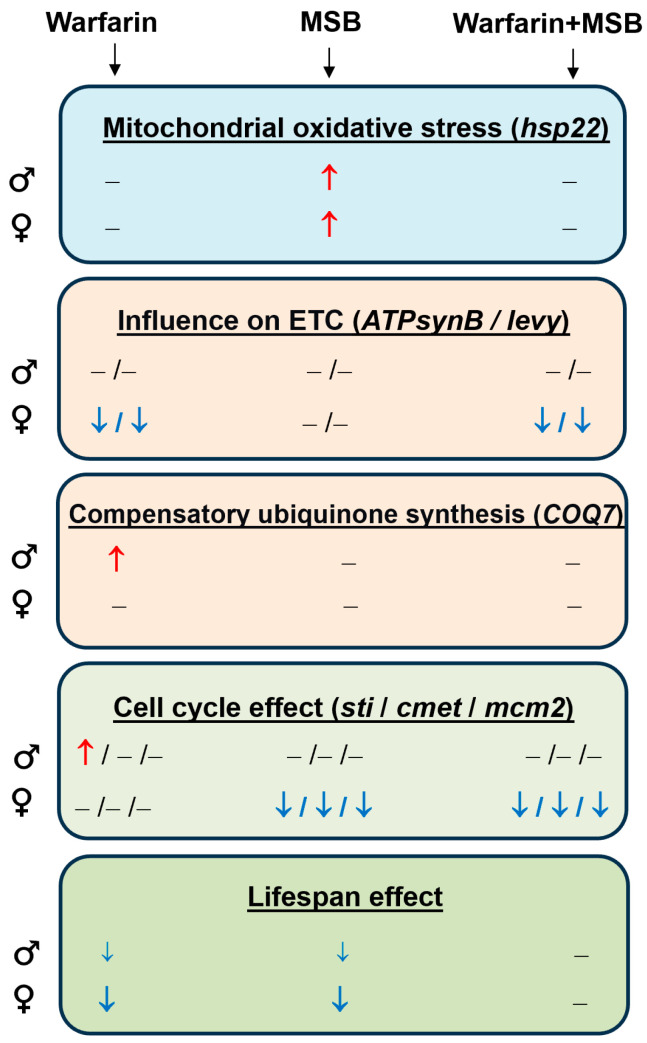
Sex-specific molecular and physiological responses to MSB and warfarin in *D. melanogaster.* ETC—electron transport chain. Red up arrows indicate increased expression, and blue down arrows indicate decreased expression relative to the control.

The transcriptional and lifespan effects of MSB and warfarin were consistently sex-specific: females exhibited reduced expression of mitochondrial and cell cycle genes and decreased lifespan upon exposure to either compound, while males remained largely unaffected (Figure 5). We attribute this sexual dimorphism to higher baseline metabolic and reproductive demands in females, which increase their vulnerability to mitochondrial stress but also enable a robust adaptive (ATP hydrolysis-based) response when the two compounds are combined. *D. melanogaster* females typically live longer but respond more strongly to metabolic disturbances during development [20]. These findings emphasize that sex is a critical biological variable in studies of vitamin K-related metabolism and mitochondrial functioning in *D. melanogaster*.

Our observation of sex-specific transcriptional and physiological responses to MSB and warfarin in *D. melanogaster* is consistent with evidence demonstrating that mitochondria from males and females may respond differently to various stressors in animals. For example, in rats, acute hypoxia during exercise impaired complex II-linked oxidative phosphorylation in heart subsarcolemmal mitochondria of females but not males, whereas males exhibited altered ADP/O ratios and increased electron transport system efficiency in brain mitochondria [21]. Similarly, sex differences in mitochondrial bioenergetics, oxidative stress susceptibility, and longevity have been widely documented. The greater sensitivity of *D. melanogaster* females to MSB and warfarin alone, coupled with their ability to benefit from the combination, mirrors findings in mammals where hormonal status (estrogen signaling) influenced mitochondrial gene expression, reactive oxygen species production, and lifespan regulation [22,23]. Thus, our results on *D. melanogaster* are in accordance with the emerging view that sex is a critical biological variable in mitochondrial toxicology and metabolic adaptation, and they support the use of flies as a model to dissect the conserved molecular mechanisms underlying sex-specific responses to vitamin K-related compounds.

## 4. Materials and Methods

### 4.1. D. melanogaster Cultivation Conditions

*D. melanogaster* wild-type strain Canton-S from the collection of the Department of Genetics, Moscow State University, was cultured at 26 °C on a standard medium consisting of raisins, semolina, yeast, agarose, and sugar. Flies were transferred to fresh medium every 15–25 days. To study the effects of MSB and warfarin on Drosophila, flies for egg laying were placed on standard medium supplemented with MSB at a concentration of 3.5 mM, warfarin at a concentration of 1 mM, or a mixture of these substances. Standard medium was used as a control. After 24 h, the parental adults were removed; the larvae developed into adults, which were collected for further analysis on the second day after eclosion.

To assess the effect of warfarin on adult survival, two-day-old males and females were placed on media containing the drug at concentrations of 1 mM and 10 mM for egg laying. The experiment was performed in six replicates. To study the effect of warfarin on egg laying, ten three-day-old females were placed for 24 h on media containing different concentrations of warfarin (1 mM, 10 mM) and on warfarin-free medium.

For the lifespan study, Drosophila were cultivated on standard medium and on media containing 3.5 mM MSB, 1 mM warfarin, or a mixture of the compounds. After eclosion, adults were transferred to standard medium; males and females were kept separately in vials, 25–30 individuals per vial. The total sample size for each treatment ranged from 35 to 85 individuals. Dead flies were counted, and flies were transferred to fresh medium every 2–3 days. Statistical analysis was performed using the log-rank test with correction for multiple comparisons with Online application for survival analysis (OASIS 2) (https://sbi.postech.ac.kr/oasis2/, accessed on 18 June 2026) [24].

### 4.2. Total RNA Extraction

Total RNA was extracted using the ExtractRNA kit (Evrogen, Moscow, Russia) according to the manufacturer’s recommended protocol. For RT-PCR experiments, 5 adults (sex considered) were placed into each tube. RNA concentration was measured using a NanoDrop2000 instrument (Thermo Fisher Scientific, Waltham, MA, USA) by recording the absorption spectrum of the RNA sample at wavelengths of 205–290 nm. The RNA solution was stored at −20 °C. To remove DNA, DNase I (RNase-free) (Thermo Scientific, USA) was used according to the manufacturer’s recommended protocol. RNA samples were stored at −20 °C.

### 4.3. Reverse Transcription

For cDNA synthesis from the extracted total RNA template after DNase I treatment, the MMLV RT kit (Evrogen, Moscow, Russia) was used with modifications. Briefly, 1 µL of random primer and 3 µL of bidistilled water were added. The mixture was incubated for 2 min in a thermostat at 70 °C and then transferred to an ice bath and cooled, and 4 µL of reverse transcriptase buffer, 2 µL of dNTPs, 2 µL of DTT, 2 µL of bidistilled water, and 1 µL of MMLV reverse transcriptase were added. The mixture was incubated for 5 min at room temperature and then for 1 h in a thermostat at 40 °C. Subsequently, it was transferred to a thermostat at 70 °C and incubated for 10 min. cDNA samples were stored at −20 °C.

### 4.4. Real-Time PCR

For real-time PCR, a reaction mixture with hot-start Taq polymerase and SYBR Green I intercalating dye (Evrogen, Moscow, Russia) was used. The reaction was performed in a DTprime Real-Time PCR System (DNA-Technology, Moscow, Russia). The following protocol was used: 5 min at 95 °C, then 40 cycles: 10 s at 95 °C, 30 s at 55 °C, and 60 s at 72 °C. A negative control with samples treated with DNase I was also included. The genes and primers used in this study are listed in Table 5. The transcription levels of the selected genes were analyzed using the ΔCt method. Expression of each gene was analyzed in 3–7 biological replicates. Expression levels of the studied genes were calculated by normalizing to the geometric mean of three reference genes Ct: *alphaTub84B*, *eIF1A*, and *EloB.*

Statistical analysis and graph plotting were performed using GraphPad Prism 9. All graphs show the distribution of the data: the maximum, minimum, median, and first and third quartiles. Kruskal–Wallis test with post hoc Dunn’s test was used to identify statistically significant differences in the gene expression levels between groups.

### 4.5. Measurement of ATP Hydrolysis Rate in D. melanogaster Mitochondria

Drosophila mitochondria were isolated according to a previously described method [25], aliquoted and frozen at −80 °C for subsequent analysis. Oligomycin, a specific inhibitor of mitochondrial F_0_F_1_-ATPase, was used to assess the purity of the mitochondrial sample preparations. According to the assessment of oligomycin-sensitive ATPase activity, the mitochondrial preparation we isolated was 94% pure as a result of isolation. The ATP hydrolysis rate was measured according to the previously published protocol using red absorbance (557/620 nm) [26]. The reaction mixture composition was: 250 mM sucrose, 5 mM MgCl_2_, 2.5 mM Hepes, 100 mM KCl, 0.1 mM EDTA, 30 µM phenol red, pH 7.4.

## Figures and Tables

**Table 1:** Results of the log-rank test for female survival curves.

Conditions	χ^2^	*p*-Value	Bonferroni *p*-Value
Control vs. MSB 3.5 mM	4.47	0.0344	0.1032
Control vs. warfarin 1 mM	8.11	0.0044	0.0132
Control vs. MSB 3.5 mM + warfarin 1 mM	1.84	0.1747	0.5242
MSB 3.5 mM vs. warfarin 1 mM	0.38	0.5393	1
MSB 3.5 mM vs. MSB 3.5 mM + warfarin 1 mM	2.01	0.1562	0.4685
Warfarin 1 mM vs. MSB 3.5 mM + warfarin 1 mM	3.22	0.0729	0.2188

**Table 2:** Results of the log-rank test for male survival curves.

Conditions	χ^2^	*p*-Value	Bonferroni *p*-Value
Control vs. MSB 3.5 mM	2.64	0.1044	0.3133
Control vs. warfarin 1 mM	5.77	0.0163	0.0489
Control vs. MSB 3.5 mM + warfarin 1 mM	3.08	0.0792	0.2375
MSB 3.5 mM vs. warfarin 1 mM	1.19	0.276	0.828
MSB 3.5 mM vs. MSB 3.5 mM + warfarin 1 mM	0	0.9576	1
Warfarin 1 mM vs. MSB 3.5 mM + warfarin 1 mM	0.86	0.3531	1

**Table 3:** Mean/median lifespan for female survival curves.

Conditions	Restricted Mean			Age in Days at % Mortality				
	Days	Std Error	95% C.I.	25%	50%	75%	100%	95% Median C.I.
Control	46.03	2.3	41.53~50.53	29.00	54.00	62.00	68.00	47.0~54.0
MSB 3.5 mM	38.11	2.14	33.92~42.31	22.00	31.00	58.00	75.00	29.0~40.0
Warfarin 1 mM	36.52	2.24	32.13~40.92	22.00	29.00	58.00	68.00	26.0~34.0
MSB 3.5 mM + warfarin 1 mM	45.1	2.09	41.01~49.19	29.00	54.00	58.00	68.00	47.0~54.0

**Table 4:** Mean/median lifespan for male survival curves.

Conditions	Restricted Mean			Age in Days at % Mortality				
	Days	Std Error	95% C.I.	25%	50%	75%	100%	95% Median C.I.
Control	55.2	1.56	52.14~58.25	54.00	58.00	62.00	75.00	54.0~54.0
MSB 3.5 mM	52.2	1.39	49.47~54.93	47.00	54.00	58.00	75.00	54.0~54.0
Warfarin 1 mM	47.15	2.83	41.61~52.69	40.00	58.00	58.00	62.00	40.0~54.0
MSB 3.5 mM + warfarin 1 mM	53.35	1.67	50.08~56.62	54.00	58.00	58.00	68.00	54.0~54.0

**Table 5:** Primer sequences used for RT-PCR analysis.

Gene	Forward (5′-3′)	Reverse (5′-3′)
*ATPsynB*	GTGGGTCTGATCACCTATC	ACTCAGATTCGATTTTATCGATC
*COQ7*	ACCCGGATAAGGAGCTGT	GATGGCAATAGCCGTCTTG
*COX5A*	AAGTACTTCAGCCGTGAGG	ACTTGTCCTTGCATCCCTC
*COX4*	ATGAACATCTTCGTGTACGA	GCACTTAGTTCTTCCACTTC
*levy*	GAGCACTCTGGTGGCTA	GGTAGTCGTACTTGACGAAC
*mcm2*	GAGTGGGTCAGTATGCTAGG	AATACGTGTTCCTTGTTGGC
*sti*	TTCTCCTGAAGCTTTTCATC	TTGCTAAAAAGTCCCTTACG
*cmet*	CTCACGTGTGAACTTTTCTG	GGAAATTAATGAGCTACGCG
*hsp22*	CTTTCACGCCTTCTTCCAC	GTGAGTTTGTAGCCATCCTTG
*eIF1A*	GGGGAAACTTCGCAAGAAG	AGCCATCCTCCACGAATG
*EloB*	AAAGACCACCATCTTCACG	CCACCTCGTTCCTGAATG
*alphaTub84B*	ATAAAAACTCAATATGCGTGAAT	GAAGGTGTTGAACGAGTC

## Data Availability

The original contributions presented in this study are included in the article. Further inquiries can be directed to the corresponding author.

## Abbreviations

The following abbreviations are used in this manuscript:

MSB	Menadione sodium bisulfite
ROS	Reactive oxygen species
VKOR	Vitamin K oxidoreductase
GGC	Gamma-glutamyl carboxylase
ETC	Electron transport chain
